# Effect of Long-Term Use of Bisphosphonates on Forearm Bone: Atypical Ulna Fractures in Elderly Woman with Osteoporosis

**DOI:** 10.1155/2016/4185202

**Published:** 2016-08-10

**Authors:** Yusuf Erdem, Zafer Atbasi, Tuluhan Yunus Emre, Gülis Kavadar, Bahtiyar Demiralp

**Affiliations:** ^1^Orthopedics and Traumatology Department, Girne Military Hospital, Kyrenia, Cyprus; ^2^Orthopedics and Traumatology Department, Ankara Military Hospital, Ankara, Turkey; ^3^Orthopedics and Traumatology Department, Memorial Hizmet Hospital, Istanbul, Turkey; ^4^Physical Medicine and Rehabilitation Department, Medicine Hospital, Istanbul, Turkey; ^5^Orthopedics and Traumatology Department, Medipol University Hospital, Istanbul, Turkey

## Abstract

Osteoporosis is a common musculoskeletal disease of the elderly population characterized by decreased bone mineral density and subsequent fractures. Bisphosphonates are a widely accepted drug therapy which act through inhibition of bone resorption and prevent fractures. However, in long-term use, atypical bisphosphonate induced fractures may occur, particularly involving the lower weight bearing extremity. Atypical ulna fracture associated with long-term bisphosphonate use is rarely reported in current literature. We present a 62-year-old woman with atypical ulna due to long-term alendronate therapy without a history of trauma or fall. Clinicians should be aware of stress fracture in a patient who has complaints of upper extremity pain and history of long-term bisphosphonate therapy.

## 1. Introduction

Osteoporosis is a common musculoskeletal disease of the elderly population characterized by decreased bone mineral density and subsequent fractures. The prevalence of osteoporosis is frequent among the subjects older than 75 years, and almost one-third of this population is affected [1]. Due to the higher prevalence, osteoporosis-related fractures gained importance as both the population of the world is expanding and life expectancy is rising [2, 3].

Although there are several treatment modalities used in osteoporosis, bisphosphonates (alendronate, risedronate, ibandronate, and zoledronic acid) are a widely accepted drug therapy which inhibit bone resorption [4]. On the other hand, long-term use of bisphosphonates may cause atypical insufficiency fractures. Bisphosphonate associated fractures commonly involve lower extremities particularly seen in femur subtrochanteric location [5–7]. However, bisphosphonate induced ulnar fractures are rarely reported in relevant literature [8–14]. Herein, we present a case of bifocal ulnar fracture that occurred twice within a period of two years.

## 2. Case Report

**Figure 1:**
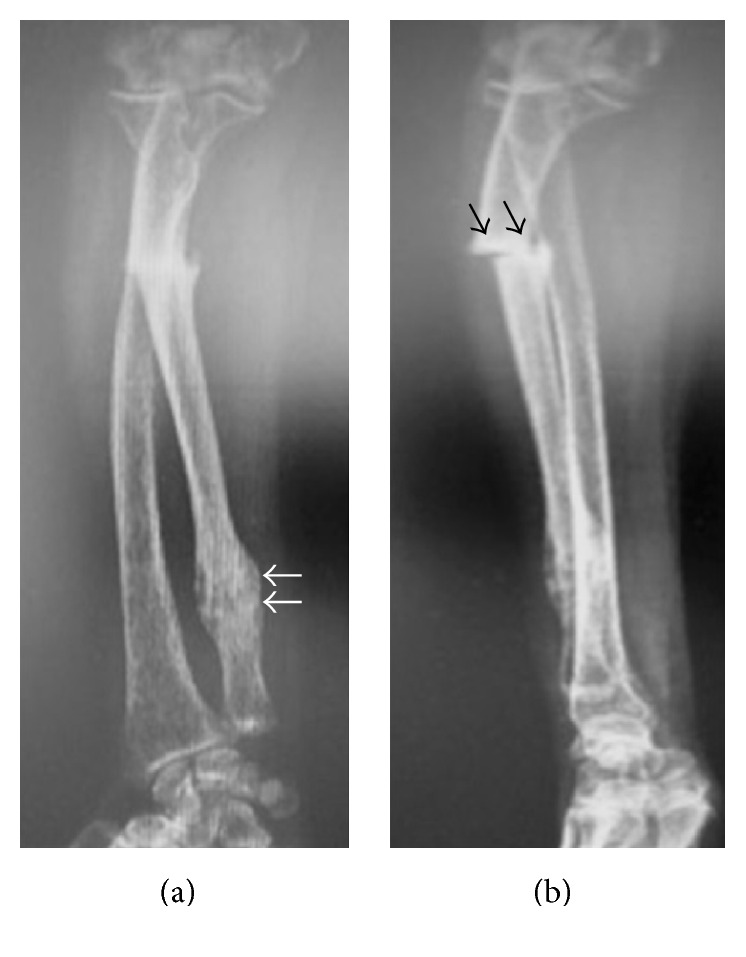
Anteroposterior (a) and lateral radiographs (b) of the patient's forearm at initial admission. Black arrows show the atypical fracture line. White arrows show the previous healed fracture.

A 62-year-old female was admitted to our outpatient clinic with pain on the ulnar side of the right (dominant) forearm which began suddenly after lifting a light object (grocery bag) seven days ago. There was no other history of trauma or fall. On examination, there was pain over the ulnar shaft on palpation, and elbow and wrist range of motion was painful. Neurovascular examination revealed no abnormality. Plane radiographic examination revealed a fracture at the proximal one-third of the ulna. Moreover, there was a hypertrophic callus formation on the distal ulna. The fracture was a transverse fracture and there was marked bony sclerosis at the fracture line (Figure 1).

**Figure 2:**
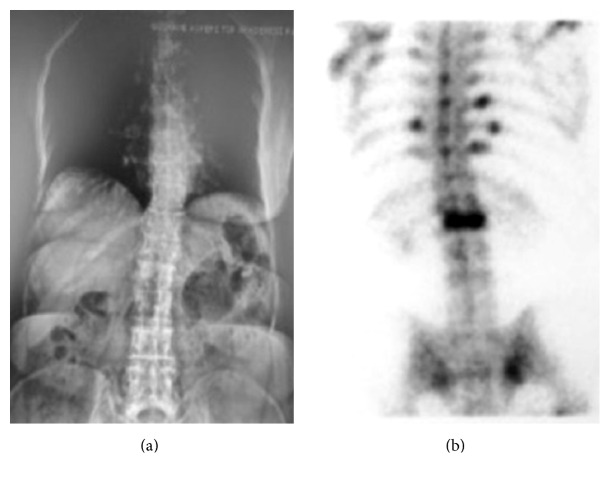
(a) Plane radiography and (b) scintigraphy showing L1 osteoporotic vertebra fracture.

Her past medical history revealed that she had type 2 diabetes mellitus, rheumatoid arthritis, and hypertension diagnosed 12 years ago. Moreover, 7 years ago she had low back pain and radiodiagnostic studies detected L1 osteoporotic vertebrae fracture (Figure 2). Osteoporosis was diagnosed based on low* T*-score in DEXA, and bisphosphonate therapy was initiated (70 mg alendronate sodium/week). She was still using bisphosphonate, approximately for a 7-year duration. Three years ago, she underwent bilateral sequential total hip arthroplasty due to marked coxarthrosis secondary to rheumatoid arthritis and used a walking cane for the duration of one year. During the rehabilitation period, when the patient was still using the walking cane, a distal ulnar stress fracture occurred and was treated with conservative treatment.

With the prolonged use of bisphosphonates, substantial amount could be accumulated over the skeletal binding sites which could cause nonunion or delayed union by conservative treatment and by being the dominant hand fracture, so aiming to enhance the early mobilization, operative treatment (open reduction and plate/screw fixation) was offered to the patient for enhancement, but the patient denied the surgical intervention. Bisphosphonate treatment was ceased and a long-arm plaster cast was applied to the patient. Laboratory blood tests including serum calcium, phosphate, alkaline phosphatase, vitamin D, thyroid, and parathyroid hormone levels were all normal to evaluate whether to use bisphosphonates after bone healing.

**Figure 3:**
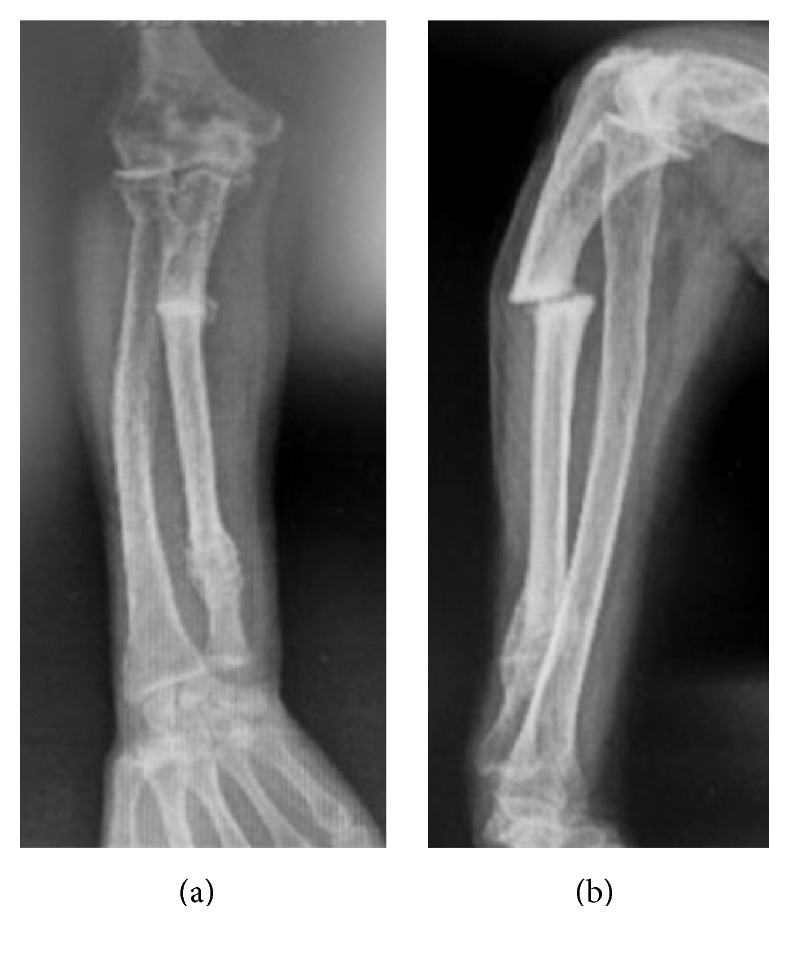
Forearm radiographs at the 6th week follow-up demonstrating the nonunion.

At the 4th week, long-arm cast was changed to short arm cast and continued for two more weeks. At the end of 6 weeks, the plaster cast was removed and the pain subsided, but there was no sign of bony union at the fracture site (Figure 3). The patient continued to deny the operative treatment. After the last visit at the 6th week, the patient was lost to follow-up.

## 3. Discussion

Either fatigue (related to overuse) or insufficiency (related to failure in bone structure) type of stress fractures is rarely seen in upper extremities [8]. In particular, ulna stress fractures are the least among them. These fractures are mostly reported in athletes. Sujino et al. and Steunebrink et al. reported bilateral ulnar fractures in a Kendo player and a weight-lifter, respectively [15, 16]. Other than overuse related to sports, ulnar stress fractures may be related to crutch use as Grace et al. have reported recently [17]. This fracture can also be accepted as overuse due to repetitive loading to upper extremity.

In current literature, there are few cases that report ulna fractures due to long-term bisphosphonate therapy [8–14]. In a recent systematic review, Tan et al. could document only seven patients with ulnar fractures secondary to long-term bisphosphonate use [8]. They emphasized that fractures due to long-term use of bisphosphonates have characteristic changes in plane radiography and common risk factors. According to their analysis, all patients were on bisphosphonate therapy for 7 to 15 years and predisposing factors included elderly females requiring walking aids. Similarly, our patient had a history of 7 years' bisphosphonate therapy and crutch use. Furthermore, dominant upper extremity was affected in most of the cases like our case. In contrast to femoral fractures, ulnar fractures occur without any type of trauma. Thus, clinicians may miss the diagnosis due to atraumatic nature of these fractures.

Neviaser et al. reported 25 bisphosphonate induced femoral fractures and analyzed the fracture pattern in these patients. 20 out of 25 patients demonstrated similar fracture patterns, namely, transverse fracture, cortical thickening, and sclerosis [7]. Similarly, Tan et al. reported that ulnar fractures are transverse, located in proximal ulna, and noncomminuted and had localized periosteal or endosteal thickening at the fracture site and generalized cortical thickening of the diaphysis [8]. Although the exact mechanisms that caused this typical fracture pattern are not clearly understood, it is believed that abnormal bone turnover results in brittle bone structure similar to osteopetrosis [13].

The treatment of these fractures can present several problems. First, there may be difficulties in fracture fixation such as proving enough primary stability. Second, fracture union cannot be achieved or delayed. If the patient is still on bisphosphonate therapy, it should be immediately stopped; thus bisphosphonates accumulation on fracture surface had been prevented to break the resistance to fracture site resorption [10–13]. Early diagnosis of undisplaced occult fractures is crucial and prophylactic surgical fixation is recommended due to difficulties in fracture site fixation. In case of ulnar fractures, open reduction and fixation should be chosen even if the fracture is nondisplaced or incomplete. Tang and Kumar reported a case of nonunion upon conservative treatment (plaster cast for 2 months) which had happened like in our case [12].

In conclusion, this case has some important messages for the readers. First, bisphosphonates should be ceased once the increase in BMD and decrease in biochemical bone turnover markers (urine cross-linked N-telopeptides of type 1 collagen, cross-linked C-telopeptides of type 1 collagen, bone-specific alkaline phosphatase, osteocalcin, and propeptide of type 1 collagen) compared to initial levels are achieved to prevent atypical fractures. We believe that patients should be individually reevaluated by using fracture risk assessment system (FRAX) before making a decision on whether a drug holiday is necessary [18, 19]. Second, clinicians should suspect a possible insufficiency fracture even in upper extremity in patients under long-term bisphosphonate therapy.
